# Untethering the Nuclear Envelope and Cytoskeleton: Biologically Distinct Dystonias Arising from a Common Cellular Dysfunction

**DOI:** 10.1155/2012/634214

**Published:** 2012-05-06

**Authors:** Nadia A. Atai, Scott D. Ryan, Rashmi Kothary, Xandra O. Breakefield, Flávia C. Nery

**Affiliations:** ^1^Neuroscience Center, Department of Neurology, Massachusetts General Hospital, East, Boston, MA 02114, USA; ^2^Center for Molecular Imaging Research, Department of Radiology, Massachusetts General Hospital, East, Boston, MA 02114, USA; ^3^Program in Neuroscience, Harvard Medical School, Charlestown, Boston, MA 02129, USA; ^4^Department of Cell Biology and Histology, Academic Medical Center (AMC), University of Amsterdam, 1105 AZ Amsterdam, The Netherlands; ^5^Ottawa Hospital Research Institute, Department of Cellular and Molecular Medicine, Ottawa, ON, Canada KIH 8L6

## Abstract

Most cases of early onset DYT1 dystonia in humans are caused by a GAG deletion in the *TOR1A* gene leading to loss of a glutamic acid (Δ*E*) in the torsinA protein, which underlies a movement disorder associated with neuronal dysfunction without apparent neurodegeneration. Mutation/deletion of the gene (*Dst*) encoding dystonin in mice results in a dystonic movement disorder termed *dystonia musculorum*, which resembles aspects of dystonia in humans. While torsinA and dystonin proteins do not share modular domain architecture, they participate in a similar function by modulating a structural link between the nuclear envelope and the cytoskeleton in neuronal cells. We suggest that through a shared interaction with the nuclear envelope protein nesprin-3**α**, torsinA and the neuronal dystonin-a2 isoform comprise a bridge complex between the outer nuclear membrane and the cytoskeleton, which is critical for some aspects of neuronal development and function. Elucidation of the overlapping roles of torsinA and dystonin-a2 in nuclear/endoplasmic reticulum dynamics should provide insights into the cellular mechanisms underlying the dystonic phenotype.

## 1. Introduction

### 1.1. Human DYT1 Dystonia

 Dystonia is the third most common movement disorder in humans, following essential tremor and Parkinson's disease. Dystonia is characterized by involuntary sustained muscle contractions that lead to twisting movements and abnormal postures [1] and comprise a heterogeneous set of clinical syndromes (for review see [2, 3]). Dystonias are generally classified on the basis of the bodily distribution of symptoms (focal, segmental, or generalized), age of disease onset (early or late), and etiology (primary or secondary). Dystonias are classified as primary when the symptoms develop spontaneously in the absence of any apparent cause or associated disease and as secondary when symptoms result from other disease states, for example Parkinson's disease or brain injury. Dystonia can be caused by dysfunction in many regions of the human brain, usually without detectable neurodegeneration. However, in a few hereditary dystonia conditions and secondary cases of dystonia, neurodegeneration may be involved.

Many primary dystonias have a hereditary component, and once the abnormal movements appear they do not remit. Twenty different genes have been implicated in various types of dystonia, many of which are inherited as autosomal dominant traits with reduced penetrance [4, 5]. Therefore, the identification of common molecular pathways that underlie multiple dystonia subtypes would improve our ability to develop new treatment strategies with wide therapeutic applications. Among the known human dystonia genes, the *TOR1A* gene, encoding the torsinA protein, is responsible for early-onset, torsion dystonia (DYT1) and has been the most studied type of dystonia [6]. Historically, DYT1 dystonia was referred to as *dystonia musculorum deformans *and thought to be inherited as an autosomal recessive condition [7, 8].

DYT1 is a severe form of hereditary, generalized dystonia [9]. Most cases are now known to be caused by a dominantly inherited in-frame GAG deletion in the *TOR1A* gene, with only a few other isolated mutations in this gene associated with a dystonic phenotype [10–12]. The GAG deletion results in loss of a glutamic acid residue in the protein product, referred to as torsinAΔ*E*. DYT1 patients are all heterozygous gene carriers, with no homozygotes harboring the mutation identified in humans to date.

### 1.2. TorsinA Findings

 Since the discovery of the *TOR1A* gene [6], a number of cellular processes have been associated with torsinA, albeit the specific molecular mechanism through which mutated torsinA leads to DYT1 dystonia remains unclear. TorsinA is predominantly localized in the contiguous lumen of the endoplasmic reticulum (ER) and the nuclear envelope (NE). However, when the mutant form of torsinA, torsinAΔ*E*, is overexpressed, it concentrates in the perinuclear space of the NE, forming inclusions in both neuronal and nonneuronal cells [13–16]. In the few studies published on neuropathology in DYT1 brains, abnormalities in torsinA localization or signs of inflammation/neurodegeneration have not been reported [17, 18]. In a small sample set, dopaminergic cell bodies in the substantia nigra appeared to be enlarged in DYT1 patients compared to normal individuals [19]. In one report, pathological abnormalities were described in brains of DYT1 patients, included ubiquitin and torsinA-positive inclusions in neurons in the brainstem, ubiquitin-positive inclusions in pigmented neurons in the substantia nigra and locus coeruleus, as well as elevated levels of tau, a protein associated with microtubules (MTs) [20]. Similar inclusions have been observed in some of the DYT1 mouse models generated by transgenic overexpression of human torsinAΔ*E* [21–23].

In mouse models of DYT1 dystonia, including homozygous knock-out of torsinA and knock-in of torsinAΔ*E*, marked morphological changes have been found in the NE of neurons [24]. These findings suggest that neurons are especially dependent on the function of torsinA in defining nuclear structure, which, in turn, may affect movement of the nucleus in relationship to the cytoskeleton during neuronal migration in developmental phases. TorsinA interacts with proteins that span the inner nuclear membrane (INM), specifically lamina-associated polypeptide 1 (LAP1) [25] and Sad1 and UNC84 domain containing 1 (SUN1) [26], as well as proteins in the outer nuclear membrane (ONM), such as the nesprins (nuclear envelope spectrin repeat) [27]. LAP1 is a type II membrane protein consisting of three variants, LAP1A, 1B, 1C, which play a crucial role in providing attachment of lamins to the INM [28]. LAP1 depletion causes similar NE abnormalities to those observed in torsinA-null cells [29]. Furthermore, the absence of SUN1 results in movement of torsinA and torsinAΔ*E* away from the NE into the ER. In contrast, LAP1 is not required for torsinAΔ*E* localization to the NE. SUN proteins have a nuclear domain that mediates interaction with lamins and an NE lumenal domain that interacts with nesprin proteins, thereby forming the “linker of nucleoskeleton and cytoskeleton” (LINC) complex [30–33]. Nesprin proteins are exclusively ONM cytoskeletal linkers mediating interactions of the nucleus with various cytoskeletal structures, such as actin filaments (AFs), intermediate filaments (IFs), and MTs, as well as associated motor proteins, such as kinesin-1/2, dynein, and dynactin [34]. Therefore, torsinA and its NE interacting proteins may have a role in modulating the link between the NE and the cytoskeleton, enabling the nucleus to negotiate its position within the cell during polarization, migration, and differentiation.

### 1.3. Murine Dystonia Musculorum (*dt*)

 Mutations in the dystonin (*Dst*) gene in mice result in a movement disorder phenotypically resembling some aspects of early-onset torsion dystonia in humans. These mice were therefore termed *dt* [35, 36]. While heterozygous *dt* mice appear to be normal throughout life, homozygous* dt* mice exhibit an early-onset phenotype. Mice are asymptomatic at birth, but by postnatal days 10–12 they begin to exhibit signs of twitching, writhing, and uncoordinated movements. The *dt *mice are typically characterized as having a sensory neuropathy with associated neurodegeneration, although motor neuron abnormalities and some brain involvement have recently been identified [37].

### 1.4. Parallels between Human DYT1 and Murine *dt* Dystonias

 Although there are many differences between human DYT1 dystonia, a dominantly inherited brain disorder with no marked neuropathology, and murine *dt* dystonia, a recessively inherited disorder primarily involving neurodegeneration in the peripheral nervous system, these forms of dystonia share common elements at the level of cellular dysfunction. A comparison of these disorders was initially confounded by the fact that aberrations in function of the human homologue of the *Dst* mouse gene are associated with skin blistering, as well as neuropathologies, and not with human dystonia [38–40].

Surprisingly, recent molecular and cellular studies on the functions of dystonin and torsinA have provided interesting molecular parallels between the potential roles of these proteins in interactions between NE/ER and the cytoskeleton. Both torsinA and the dystonin-a2 neuronal isoform have been reported to interact with nesprin-3*α* [27, 41] located in the ONM of the NE [42]. Nesprin-3*α* interacts with the multifunctional cytoskeletal linker plectin, which consists of various isoforms that facilitate interaction with IFs [42]. Plectin is highly expressed in various cell types and plays a major role in cellular cytoskeletal organization and dynamics [43]. Plectin interaction with nesprin-3*α* serves to link the NE membrane and IFs, which in turn are linked to AFs and MTs through members of the spectraplakin protein family [42, 43].

Consolidation of the existing literature on subcellular localization, protein interactions, and loss of function behavioral correlates led us to speculate that torsinA and dystonin-a2 may both participate in protein complexes associated with membranes of the NE/ER and the cytoskeleton. In this paper, we present current knowledge of torsin and dystonin proteins, including their functional similarities and differences, in order to highlight common mechanisms that may underlie different forms of dystonia through disruption of the link between the NE/ER and cytoskeleton that may impair neuronal development and function.

## 2. Torsin-Related Proteins

**Figure 1:**
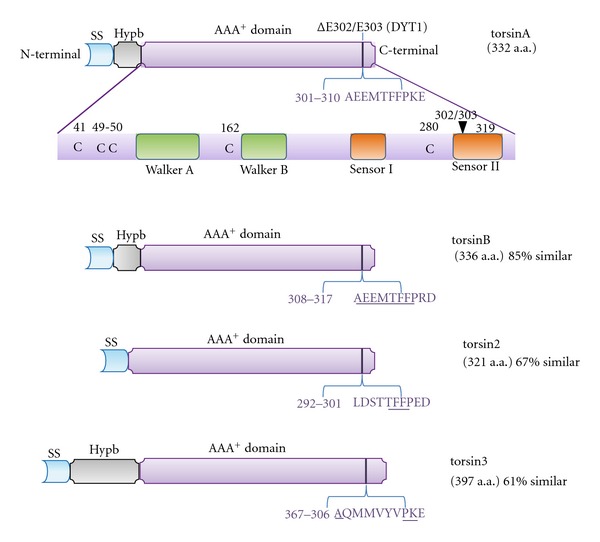
Schematic diagram of the AAA^+^ torsin protein family. The key features of torsinA, torsinB, torsin2, and torsin3 are shown, including the signal sequence (SS; turquoise), hydrophobic domain (Hypb; grey), and AAA^+^ domain (purple). The AAA^+^ domain of torsinA, illustrated in more detail, consists of Walker A/B (green) motifs, sensor I/II (orange) motifs, and six conserved cysteines (C). Also, the total number of amino acids (a.a.) for each protein and the overall percentage similarity with torsinA are indicated. In torsin2, the Hydb domain is absent and torsin3 has a longer Hydb domain. The 10 a.a. region in torsinA in which the glutamic acid deletion underlying DYT1 dystonia occurs is shown in detail. Conserved a.a. residues in this region in other members of the torsin family are underlined (figure revised from Jungwirth et al. [26] and Zhu et al. [54]).

Torsin proteins are members of the AAA^+^ superfamily of ATPases [44]. Most AAA^+^ proteins form oligomeric complexes (typically six subunit rings) obtaining energy from ATP hydrolysis and acting as chaperone-like modules [45]. AAA^+^ proteins are involved in different cellular processes such as protein folding, membrane trafficking, vesicle fusion, and cytoskeleton dynamics [45–47]. Other activities associated with AAA^+^ proteins include peroxisome biogenesis, assembly of mitochondrial proteins, cell cycle control, mitotic spindle formation, cytoskeleton interactions, vesicle-mediated secretion, signal transduction, and transcriptional regulation [48–51]. The AAA^+^ domain of torsinA contains the Walker A motif (also known as the P-loop-ATP binding domain), the Walker B motif (ATP hydrolysis), and the ATP sensing motifs (sensor I and sensor II) [52, 53], and six prominently conserved cysteine residues (Figure 1). Their location in the lumen of the NE/ER and cysteine residues in the AAA^+^ domain distinguish these torsin family members from other AAA^+^ proteins [52, 54].

Two structural models of human torsinA are based on the crystal structure of orthologues in bacteria, ClpA [52] and ClpB [53] proteases, also members of the AAA^+^ ATPase superfamily that form homohexameric structures. Both structures suggest that torsinA is composed of two subdomains, a N-terminal *α*/*β* subdomain and a C-terminal *α*-helical subdomain. The latter subdomain is the region containing mutations in DYT1 dystonia patients [6, 10, 12]. The ΔGAG deletion associated with most cases of DYT1 dystonia removes a glutamic acid residue from an *α*-helix in the C-terminal subdomain [53]. Although mutant torsinA is presumed to be able to associate with wild-type torsinA subunits in six subunit rings, its altered structure may serve to decrease activity of the hexamer [55]. ATP-dependent Clp proteases are involved in nonlysosomal removal of damaged or misfolded proteins in bacteria [56, 57]. In mammalian cells mutant or misfolded proteins in the ER are eliminated by ER-associated degradation (ERAD) with torsinA facilitating exit of proteins to the cytoplasm where they are degraded by the ubiquitin-proteasome system [58]. Curiously, ClpA/ClpB and most members of the AAA^+^ protein family present a Walker A consensus GxxGxGK [T/S] motif, which contains an essential lysine that supports binding of nucleotides, whereas torsinA contains a noncanonical Walker A sequence, GxxGxGKN [47]. When the threonine in the Walker A motif was replaced with asparagine in the ClpB protease, so as to make it more similar to torsinA, the ATPase activity was partially inhibited. However, this did not affect the ability of ClpB to form hexamers. The noncanonical Walker A sequence introduced into ClpB induced preferential binding of ADP rather than ATP and reduced its chaperone activity *in vitro* and *in vivo* [47]. This suggests that torsin proteins might utilize distinct mechanisms to couple the ATPase cycle with their substrate-remodeling activity.

### 2.1. The Four Torsin Proteins in Mammals

Four genes encoding torsin family members have been identified in mammals: torsinA, torsinB (85% similar to torsinA), torsin2, and torsin3 (67% and 61% similar to torsinA, resp.) [59] (Figure 1). All four proteins share the core AAA^+^ domain [52, 53, 60]. TorsinA and torsinB genes (*TOR1A* and *TOR1B*, resp.) are adjacent to each other in opposite orientations on chromosome 9q [59]). The torsin2 gene (*TOR2*) is located on the same chromosome in nearby region. These three torsin genes (*TOR1A*, *TOR1B*, and *TOR2*) possess five exons. In contrast, the torsin3 gene (*TOR3*) is located on chromosome 1q and has six exons. TorsinA has been the most extensively studied due to its role in DYT1 dystonia. The glutamic acid deletion at position 302/303 in exon 5 of torsinA is responsible for about 80% of cases in the Ashkenazic Jewish population and about 40% in the general population. Five other variations have been found that change the amino acid sequence of torsinA: F205I in exon 3 [11] with the rest in exon 5, including R288Q [12], a 4 bp deletion that causes a frameshift and truncation starting at residue 312 [61], and an 18 bp deletion (ΔF323 − Y328) [10], but the pathogenicity of these sequence variants has yet to be established. In addition, there is a polymorphism in the coding sequence for residue 216 encoding aspartic acid (D) in 88% and histidine (H) in 12% of alleles in control populations [10], with the D216H polymorphism influencing the penetrance of DYT1 dystonia [62].

TorsinA is a 332-amino-acid protein that contains an N-terminal ER signal sequence (SS) and a 20-amino-acid hydrophobic region followed by a conserved AAA^+^ domain (Figure 1). The SS and glycosylation state of torsinA are consistent with its location in the ER/NE, with the hydrophobic N-terminal sequence apparently retaining it in this location [13, 63–65]. However, with the exception of torsinB, which has very similar characteristics to torsinA [63, 66], the subcellular localization of torsin2 and torsin3 remains poorly characterized. It was recently reported that digestion of glycosylated residues reduces the molecular weight of all four torsins in whole-cell preparations [60], indicating that all reside in the ER or at least pass through that compartment and may have overlapping functions, as do torsinA and torsinB [29].

Although torsinA is widely expressed in human tissue, it appears to have its most critical role in the central nervous system (CNS), where it is present at high levels during development [67, 68]. In adult brains, high levels of torsinA expression are found in specific neuronal populations, such as dopaminergic neurons in the substantia nigra pars compacta, interneurons in the striatum, and neurons in the cerebral cortex, thalamus, hippocampus, cerebellum, midbrain, pons, and spinal cord [19, 69–73]. TorsinA has been implicated in several cellular pathways including NE integrity [14–16, 24, 26, 29], cytoskeleton organization [27, 74–76], nuclear polarity [27] (as well as the nematode homologue, OCC-5 [77]), chaperone functions [78–82], degradation of misfolded proteins [58, 83], ER stress sensitivity [58, 84–87], dynamics of secretory and synaptic vesicles [88–92], and modulation of dopamine (DA) neurotransmission [93–107].

The second most investigated torsin protein, torsinB, is highly expressed in the lung, placenta and testis, with low levels in the brain [29, 60, 63]. Immunoprecipitation studies indicated that torsinA and torsinB associate with each other [63] and have a similar distribution in nonneuronal cells [29, 60, 66]. Mutations or other pathologies in human disease have not been reported for torsinB. Similar to torsinA, torsinB expression is temporally and spatially regulated, with torsinB expression in the developing brain peaking after that of torsinA [68, 108]. The low expression of torsinB relative to torsinA in the brain and the high expression of torsinB in other tissues may explain why the brain is selectively affected by mutant torsinA and why nonneuronal tissue are protected from torsinA dysfunction in DYT1 patients [29].

The other mammalian torsin members, torsin2 and torsin3, share the core AAA^+^ domain and the functionally important region affected by the DYT1 mutation (Figure 1) [52, 53]. Torsin3 contains an extended amino terminus, and torsin2 lacks a specific Hypb between the SS and the AAA domain (Figure 1). To determine whether other torsin family members can change their location in cells, a mutation in the Walker B motif (E171Q, ATP-bound state) of the AAA^+^ domain was generated in different members. In torsinA this mutation leads to a substrate-trap state with defects in ATP hydrolysis and accumulation of torsinA in the perinuclear region [16]. Mutated Walker B motifs in torsinB (E178Q) and torsin2 (E162Q) also led to movement out of the ER and into the NE region, while that in torsin3 (E236Q) did not change its location, which remained predominantly in the ER. In contrast to other torsin family members, torsin3 is an interferon-regulated protein [109] and may have distinct biological function(s). Notably, three of four torsin family members (torsinA, torsinB, and torsin2) operate within the NE, making them candidates for involvement in nuclear-cytoskeleton links.

### 2.2. TorsinA and Its Nonmammalian Orthologues

Besides ClpA and ClpB, which are the torsinA orthologues found in bacteria, yeast, and plants [47], other coding sequences related to the *TOR1A* gene have been found in nonmammalian organisms, including *Drosophila *(Torp4a, dtorsin), nematodes (tor-1, tor-2, and OOC-5) and zebrafish (torsinC) [55].

*Drosophila *may be a viable model for providing insights into human dystonia. Loss of dtorsin or expression of human mutant torsinA, torsinAΔ*E*, leads to abnormal motor behavior in *Drosophila *larvae [107, 110]. Interestingly, dtorsin-null flies have decreased levels of DA and movement can be normalized by feeding DA to larvae or by expressing human torsinA [107]. Dtorsin is 34% identical to human torsinA and is predominantly localized in the ER, but also found in the NE. Although neural degeneration caused by the loss of torsin function has not previously been reported in other organisms, downregulation of dtorsin resulted in increased age-related retinal degeneration in *Drosophila* [80]. In parallel, overexpression of dtorsin protected the retina from degeneration.

Three torsin-like gene products are predicted in the nematode *Caenorhabditis elegans* (*C. elegans*) (tor-1, tor-2, and OOC-5). Ectopic overexpression of either tor-2 and OOC-5 or human torsinA resulted in a reduction of polyglutamine repeat-induced protein aggregation in *C. elegans*, supporting a chaperone function [94]. Moreover, mutant forms of tor-2 were unable to ameliorate aggregation, and tor-2 and ubiquitin colocalized at sites of protein aggregation. The OOC-5 protein is also located in the ER/NE, and loss of OOC-5 disrupts nuclear rotation during early embryogenesis [77]. This nuclear rotation is mediated by cytoskeletal links between the nuclear centrosome complex and the cortical surface of the embryo [111]. In parallel, absence of torsinA interferes with the positioning of nuclei within migrating mouse fibroblasts, thus delaying initiation of migration [27]. It has also been hypothesized that the sensor II motif in torsin proteins (Figure 1) acts as a redox-regulated sensor. The oxidation of the cysteine in the sensor II motif of OOC-5 disrupts its function [52], and, in parallel, the redox state of this motif in torsinA influences its association with binding partners, LAP1 and lumenal domain like LAP1(LULL1) [54]. Both ATPase activity and protein binding to torsins are regulated by the redox state of the ER, consistent with their proposed role as ER chaperone proteins involved in processing of membrane proteins and in the ER stress response [58, 86, 88, 93].

## 3. Dystonin and the Plakin Family of Cytoskeletal Linker Proteins

The eukaryotic cytoskeleton comprises three cytoskeletal networks that include MTs, AFs, and IFs. These networks rely upon cytoskeletal cross-linking proteins, known as plakins, such as dystonin, for maintenance of their functional integrity and interactions. Cytoskeletal linker proteins are critical regulators of cellular processes ranging from vesicular transport and maintenance of organelle integrity to mitosis and cell death [112]. As such, impairment of cytoskeletal-linker function impacts cell viability, development and a variety of cellular functions and has been associated with several organelle-specific stress pathways [41, 113, 114].

### 3.1. Plakins as Cytoskeletal Linkers

Members of the plakin family of cytoskeletal proteins are critical in maintaining the function and integrity of tissues. For example, they link junctional complexes between cells (desmosomes and hemidesmosomes) with the cytoskeletal network within the cells (for review see [114]). A common structural element conserved in most family members is the plakin domain, comprising six antiparallel segments arranged in an *α*-helical fashion [115]. Numerous plakin proteins have been identified in mammalian central nervous system (CNS), including dystonin (also known as bullous pemphigoid antigen 1 (BPAG1)) [8, 116], MT-actin cross-linking factor 1 (MACF1), (also known as actin-cross linking factor 7 (ACF7)) [112, 117–119], plectin (isoforms 1a, 1b, and 1c, 1e, 2a, and 3a) [112], desmoplakin and periplakin [120]. Both plectin and desmoplakin have been demonstrated to interact directly with IF [121]. Plectin is one of the most versatile and ubiquitously expressed plakins. The association of plakins with IFs changes depending on the phosphorylation state of serine residues in the carboxy-terminal segment of the plakin, suggesting that this interaction may be regulated within cells [112]. Recently, it has been suggested that plectin serves as a nucleation and assembly center for the *de novo* formation of IFs networks and acts in turnover of focal adhesions [122]. The abundance of spectrin repeats (SRs), as found in several plakin family members, has resulted in their further subclassification as spectraplakins, namely, dystonin and MACF1 (mammalian), short stop (*Drosophila*), and variable abnormal morphology (VAB; *C. elegans*) [115, 123]. Mutations in plakins are associated with a variety of neurological disorders in humans [39, 124–126], while in animals only a mutation in *Dst* results in a neurological phenotype. Extensive structural homology exists among mammalian spectraplakins, including the SR domains, which act as docking stations for cytoskeletal elements [127], the actin-binding domain (ABD), and MT binding domain (MTBD) [115, 116, 118, 128, 129]. The multidomain nature of these proteins facilitates their function in linking cytoskeletal elements and bridging them with junctional complexes. The cellular localization and predicted activity of dystonin implicate it in functions of the peripheral sensory and motor nervous system.

### 3.2. Dystonin Isoforms Generated by Alternative Splicing

**Figure 2:**
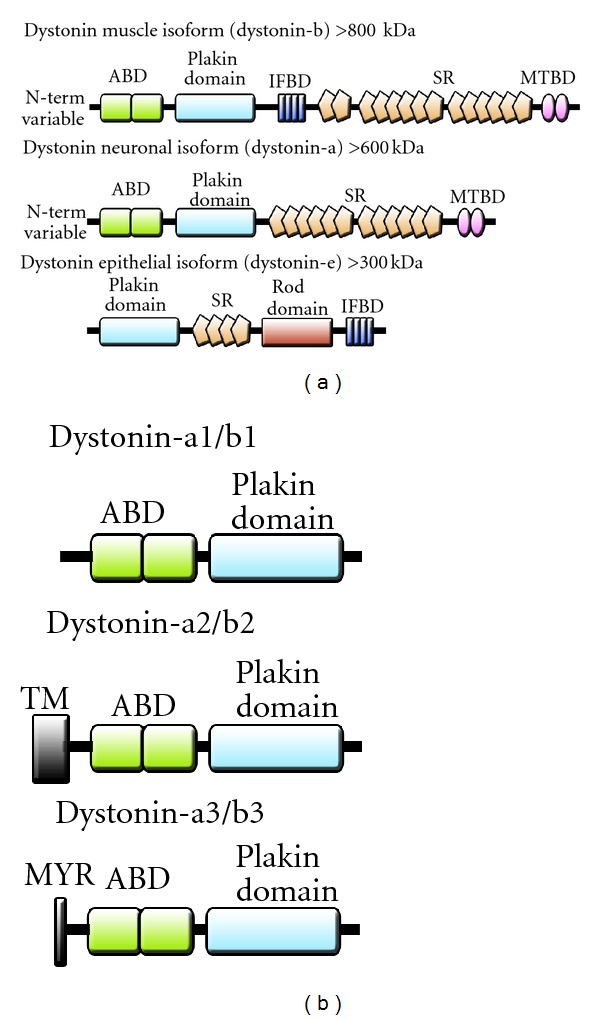
Structural representation of dystonin protein isoforms. (a) Illustration of the modular domain structure of tissue specific dystonin isoforms. Three tissue-specific isoforms have been identified; muscle (dystonin-b), neuronal (dystonin-a), and epithelial (dystonin-e). (b) Dystonin-a and b isoforms vary at the N-terminus, resulting in three distinct isoforms. The isoform 1 variant possesses a sequence coding for an ABD at the N-terminus. The isoform 2 variant contains sequence coding for a highly conserved N-terminal TM domain, while the isoform 3 variant contains sequence coding for a conserved MYR motif.

The *Dst* is a large gene (~400 kb) in mice and gives rise to three tissue-specific dystonin protein isoforms, namely, dystonin-e (epithelial isoform, ~315 kDa), dystonin-b (muscle isoform, ~834 kDa), and dystonin-a (neuronal isoform, ~615 kDa) (Figure 2(a)) [8, 128, 130, 131]. Dystonin-e serves as an auto-antigen in the skin blistering disease, bullous pemphigoid (BP) in humans and a mutation affecting this isoform results in a similar defect in mice. Loss of function of dystonin-a is believed to be causal in *dt* sensory/motor neuropathy in mice [8, 37, 131–133]. Three neuronal isoforms of dystonin-a are generated by alternative splicing, namely, dystonin-a1, dystonin-a2, and dystonin-a3 [134] (Figure 2(b)). These isoforms share an ABD sequence, an extensive coiled-coil region, and a C-terminal MTBD allowing for interactions with actin and MTs, thus facilitating their function as cytoskeletal linkers [128]. They differ on the basis of unique N-terminal regions that dictate their subcellular location. Specifically, dystonin-a1 possesses a short N-terminal domain that includes an ABD localizing it to AFs, dystonin-a2 possesses a transmembrane (TM) domain localizing it to the NE and perinuclear membranes, and dystonin-a3 possesses a putative MYR motif, aiding in anchoring it to the plasma membrane (Figure 2(b)) [135, 136]. The unique TM domain of dystonin-a2 distinguishes it from other spectraplakins. While other spectraplakins link neurofilaments (NFs) to MTs, AF or junctional complexes, dystonin-a2 links AFs and MTs with membranes of the ER, Golgi-apparatus and nucleus. Moreover, mutations in dystonin-a2 lead to induction of ER chaperone proteins and the unfolded protein response, which are believed to contribute to degeneration of both sensory and motor neurons [8, 37, 41, 137].

### 3.3. Dystonin Function in Neurons

Spectraplakins associate with MTs via two conserved domains: the growth arrest-specific 2- (Gas2-)related domain and its adjacent carboxy terminal tail region [118, 129, 138]. While the Gas2-related domain aligns along MT shafts and promotes MT stabilization [129, 138], the carboxy terminus of spectraplakin family members MACF1, and short stop bind to the MT plus-end-binding protein 1 (EB1) and localize at growing MT ends [139–141], establishing spectraplakins as putative plus-tip-interacting proteins. Direct interactions between EB1 and dystonin have not been reported, but the conserved modular domains between dystonin-a2 and MACF1 suggest that dystonin may be involved in MT polymerization. Both dystonin-a2 and MACF1b have been found in the Golgi apparatus, and overexpressed dystonin-a2 colocalizes with the *cis*-Golgi protein GM130, suggesting a functional role in Golgi apparatus-mediated MT nucleation [119, 136, 142]. Other MT-associated proteins, such as MAP1B and clathrin, have been shown to promote MT polymerization and stability when localized to the Golgi apparatus [143, 144]. Dystonin-a2 has been shown to interact with both MAP1B and clathrin via the modular plakin domain [145]. Plus-end MT polymerization from the Golgi apparatus toward the leading edge of the cell supports a role in cell migration [146, 147], and mutations in MT-associated proteins, including MAP1B and MACF1 cause defects in neuronal migration and cortical development [125, 148, 149]. Although no developmental migration defects have been reported in *dt* neurons to date, such deficits have been noted in epithelial cells lacking the smaller epithelial isoform (dystonin-e) [138].

Spectraplakins have also been implicated in the ultrastructural organization of organelles. For example, the organization of the Golgi apparatus complex is dependent on MACF1b, which shares a similar domain structure with neuronal dystonia-a2 [118, 119]. Chemical dispersion of the Golgi apparatus results in redistribution of MACF1b to the ER, while reduced levels of MACF1b induce Golgi apparatus fragmentation. Localization of MACF1b to the Golgi apparatus is mediated by its N-terminal plakin domain [119]. This domain is conserved in dystonin-a2 and oriented adjacent to the TM domain that positions the protein along perinuclear membranes [41, 136]. As such, dystonin-a2 appears to be a critical modifier of perikaryal structures and may influence functions of the ER, Golgi apparatus, and NE. Compromised organization of the ER, Golgi apparatus, and MT network would no doubt influence protein processing through the secretory pathway. Molecular interaction studies have implicated dystonin in regulation of the dynein motor complex through interaction with dynactin and the endosomal vesicle protein, retrolinkin [150, 151]. Defective fast axonal transport has also been reported in the sciatic nerves of phenotypic *dt^27J^* mice in both orthograde and retrograde directions [152]. Indeed, cytoskeletal linkers have a general role in mediating axonal transport by regulating organelle organization and movement in addition to stabilizing the cytoskeleton. Collectively, these findings show that functional impairment of sensory and motor neurons can result from defects in cytoskeletal mediated intracellular trafficking.

## 4. Studies in Humans and Mice Models with Mutations in *TOR1A* and *Dst*

### 4.1. Human DYT1 Dystonia: A CNS Movement Disorder

Dystonia has been considered to be primarily a disorder of the basal ganglia since patients with secondary dystonia commonly exhibit lesions within structures associated with the basal ganglia, including caudate, putamen, globus pallidus, and thalamus [153–155]. However, substantial evidence also implicates cerebellar and brainstem pathology in cases of primary and secondary dystonia [3, 156, 157]. Hypermetabolic signals within premotor cortex and cerebellum have been found in patients with primary dystonias, including hemidystonia, exercise-induced paroxysmal dystonia, and DYT1 dystonia [158–161]. Symptoms of dystonia have also been reported in familial forms of ataxia, where the degeneration appears to be limited to the cerebellum and brainstem [162]. Although many interconnected brain regions can be affected in human dystonia, there does not appear to be any pathophysiology in peripheral sensory or motor neurons. This stands in contrast to the *dt* dystonic motor syndrome in mice where the peripheral nervous system is primarily affected with marked pathology. It has been speculated that the high expression of other members of the plakin family in the brain, MACF1 in particular, may compensate for dystonin loss in the CNS of *dt* mice [119, 125, 163].

Genetic mouse models of DYT1 dystonia have been created to better understand the neurobiological basis of dystonia (Table 1). However, mutations that result in dystonia in humans do not generate an obvious dystonic phenotypic in mice. Mouse models of dystonia also do not show any obvious evidence of neuronal loss, although in some models the levels of striatal DA and its metabolites were reduced and animals showed some motor abnormalities (Table 1), such as hyperactivity, circling behavior, deficits in beam walking, and reduced motor learning [21, 97, 104, 164, 165]. Some models of dystonia in rats and mice implicate abnormal cerebellar signaling, including Purkinje cells and deep cerebellar nuclei [166–170]. In addition, selective elimination of the cerebellar output in some mouse and rat models served to block the dystonic symptoms [171–174].

### 4.2. Dystonia Musculorum: A Peripheral Nervous System Movement Disorder in Rodents

The *dt* mouse arose spontaneously as a recessively inherited mutation with a severe movement disorder that appeared to resemble dystonia in humans [35]. This mouse disorder develops as a progressive ataxia, due to the degeneration of the sensory neurons, and appears similar to generalized dystonia with twisting movements of the neck, paddling motions of the limbs, and abnormal posturing of limbs and trunk. The disease progresses rapidly and *dt* mice die in the third week of life of unknown causes [8, 177]. Accumulation of NF tangles within sensory neurons of *dt* mice is a pathological feature of the disease [36, 179–182]. In addition, an abnormal accumulation of hyperphosphorylated NFs is found in the perikarya and proximal regions of axons of spinal motor neurons [36]. Peripheral *dt* pathologies have been documented in motor neurons, skeletal muscle, and Schwann cells, but degeneration is most prominent in dorsal root ganglion sensory neurons [36, 37, 183, 184]. Interestingly, *dt* mice also exhibit lesions within several brain regions, including basal ganglia and cerebellum [37, 185, 186]. Several *dt* mouse models exist through spontaneous mutations (*dt^27J^*, *dt^Alb^*), chemically induced mutations (*dt^37J^*, *dt^33J^*), targeted alleles (*dt^tm1Efu^*), and transgenic insertions (*dt^Tg4^*) [130, 131] (Table 1). While only three *dt* mutations (*dt^Tg4^*, *dt^tm1Efu^*, and *dt^Alb^*) have been characterized at the DNA level, *dt^Tg4^* and* dt^27J^* are allelic but do not complement [113, 132, 137]. While it is unresolved as to whether loss of a single isoform or combination of isoforms is responsible for neuronal loss, defects in nucleoskeletal/cytoskeletal function are paramount in these *dt* models.

### 4.3. Dystonin-Related Disorders in Humans

In humans, dystonin-e was initially characterized under its pseudonym BPAG1e as a major autoantigen in the skin blistering disease, BP [38]. In keratinocytes, dystonin-e links keratin-containing IFs to hemidesmosomes [187]. Mutation of the plakin family member plectin results in a similar phenotype in humans termed epidermolysis bullosa simplex [188, 189]. The original characterization of dystonin-e-null mice described similar skin defects to those observed in humans but, unexpectedly, also showed severe neurodegeneration and dystonic symptoms [137]. While presentation of dystonin-e autoantigen in humans does not result in neurological deficits, BP patients have been reported to have an increased incidence of certain neurological diseases, such as multiple sclerosis, amyotrophic lateral sclerosis, Parkinson's disease, Alzheimer disease, and cerebral stroke [190–197]. While the reasons behind this correlation are unknown, it is interesting to speculate that mutations that affect other dystonin isoforms, such as dystonin-a may contribute to neurological symptoms observed in BP patients [197]. Indeed, in the reverse situation, an autoimmune response initially directed against neuronal dystonin-a triggered a secondary autoimmune response against dystonin-e based on their sequence homology [198–200]. Furthermore, in one human subject with a translocation in the human dystonin gene (*DST) *that specifically disrupted dystonin-a and -b isoforms, the patient presented with a profound delay in cognitive and motor function, as well as visual deficiency [39]. Curiously, no defect in epithelial tissue was observed, consistent with unaltered expression of the dystonin-e isoform. Thus, while no mutations in *DST* have been identified in humans, haploinsufficiency or altered expression can result in phenotypic abnormalities with some similarities to the inherited disorder in mice.

## 5. Similarities between Cellular Functions of TorsinA and Dystonin-a2

### 5.1. Link between NE/ER and Cytoskeleton

Mutations in both mouse *Dst* and human *TOR1A* genes result in aberrations of NE/ER morphology. The homozygous knock-out and knock-in torsinAΔ*E* mouse models of DYT1 dystonia show abnormalities in structure of the NE in embryos, largely confined to CNS neurons [25]. *dt* mice also manifest nuclear eccentricity and loss of chromophilic material from around the nucleus in neurons in the red nucleus of the rostral midbrain and discrete regions of the striatum, likely due to perinuclear swelling and disorganization of the rough ER [35, 41, 179, 185, 201]. Nuclear eccentricity has been reported for several *dt* alleles and correlates with disease onset [37, 41]. Furthermore, mutation in dystonin-a2 results in accumulation of AFs around the nucleus and Golgi apparatus [136], which is in line with human DYT1 fibroblast in which has been shown nuclear accumulation of IF protein, vimentin [75].

In addition, torsinA and dystonin proteins have been attributed with roles in ER organization and ER stress. Overexpression of wild-type torsinA has no marked effect on its ER distribution as compared with endogenous levels of torsinA, showing the typical reticular pattern throughout the cell body. In contrast, torsinAΔ*E *overexpression causes torsinA immunoreactivity to accumulate in the NE region [13, 15, 16]. Furthermore, overexpression of a truncated form of torsinA (torsinA313-332) initiates changes in ER macrostructure, resulting in a vacuolar distribution of both torsinA and protein disulfide isomerase (PDI) [58]. No TM proteins in the ER have been identified that interact with both torsinA and the cytoskeleton. However, protein processing in the ER depends on dynamic movement of membranes mediated by the cytoskeleton, especially in neurons [202]. While an association between torsinA and dystonin has not been demonstrated, the TM domain of dystonin-a2 makes it an interesting candidate in this respect. Both proteins localize to the ER and NE and associate with nesprin-3*α*, as well as influence the structure of the NE and ER membranes, suggesting that these two proteins participate in common functional pathways.

Studies in *dt^Tg4^* mice have implicated mutations in *Dst* with ER-stress induction, based on upregulation of protein chaperones, PDI and binding immunoglobulin protein (BiP), also known as 78 kDa glucose-regulated protein (GRP-78) upon phenotypic onset of dystonia [41]. This suggests the possibility that disruption in the link between the ER and the cytoskeleton may initiate a proapoptotic signalling cascade in neurons of *dt *mice initiated by ER stress. Similarly, torsinA-deficient mouse embryonic fibroblasts (MEFs) have increased ER stress, as compared to torsinA wild-type MEFs as detected by elevated BiP levels [87]. Furthermore, DYT1 fibroblasts elicit an ER stress response at lower concentrations of stress-inducing drugs as compared to fibroblasts from controls, and wild-type torsinA can reduce ER stress caused by ER protein overload in nematodes [58]. Recently, torsinA was found to coimmunoprecipitate with ERAD components, including Derlin-1, p97, and VIMP (VCP-interacting membrane protein), and downregulation of torsinA or expression of mutant torsinA compromised the cell's ability to eliminate mutant proteins from the ER [58]. Interestingly, torsinA and p97 have been found in inclusion bodies in brain and peripheral nerves of patients with a variety of neurodegenerative diseases, as well as dystonia suggesting their involvement in elimination or sequestration of toxic proteins [203–206]. Consistent with torsinA function in ERAD, it has also been found to be involved in quality control of *ε*-sarcoglycan (SGCE) processing [83], with SGCE expressed at high levels in the brain [207] and mutated in human myoclonic dystonia [208]. The involvement of torsinA in the ERAD pathway may help to explain the increased sensitivity of cells to ER stress following loss of or mutation in torsinA, with ER stress possibly contributing to onset of dystonia in DYT1 carriers [58, 86].

### 5.2. Nesprin-3*α*: A Common Associated Partner of Dystonin-a2 and TorsinA

**Figure 3:**
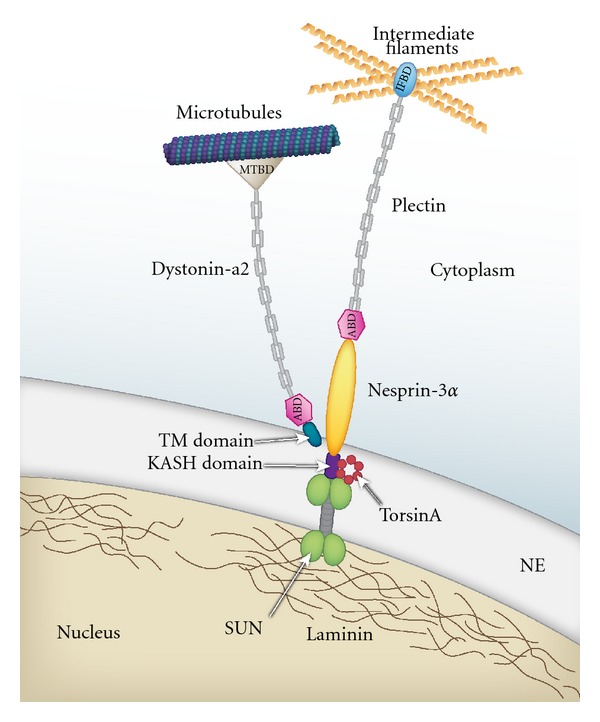
Hypothesized model for torsinA, dystonin-a2, and their common associated partner, nesprin-3*α*. This schematic represents the associations between torsinA and dystonin-a2 with nesprin-3*α*, a type II TM protein in the ONM that contains a C-terminal KASH domain within the lumenal space of the NE. Nesprin-3*α* interacts with SUN proteins (green and gray), which span the INM of the NE, through its KASH domain (purple). TorsinA (red hexameric circles) associates with nesprin-3*α* and regulates its interaction with SUN proteins. The N-terminus of nesprin-3*α* binds to the plakin family member, plectin, which in turn links to IFs through its IF-binding domain (IFBD; blue). Dystonin-a2 has a TM domain (dark blue) at the N-terminus, which embeds in the ONM of the NE and associates with nesprin-3*α*. Like plectin, dystonin-a isoforms are members of the plakin protein family and are characterized by SR domains indicated by the “chain shape” (gray) and an ABD (pink) in the N-terminal region. The dystonin-a2 isoform also possesses a C-terminal MTBD through which it associates with microtubules. Collectively, these proteins function to bridge the NE and ER with the three major cytoskeleton networks present in eukaryotes.

Nesprin proteins are evolutionarily conserved nuclear TM proteins that couple the NE with the cytoplasmic cytoskeleton (directly to MTs and AF and indirectly through plakins to IFs) [209]. Nesprin proteins possess a conserved 50 to 60 residue C-terminal KASH (Klarsicht, Anc-1, Syne Homology) domain featuring a single TM segment followed by a short lumenal sequence. To date four nesprin proteins have been identified [210]: nesprin-1 (also known as Syne1, Myne-1, and Enaptin), nesprin-2 (also known as Syne2, Myne-2, and NUANCE), nesprin-3 and nesprin-4. For nesprins-1 and -2, the primary transcripts encode several alternatively spliced isoforms [170]. The largest of these, nesprin-1 Giant (Nesp1G; 1,000 kDa) as well as nesprin-2 Giant (Nesp2G; 800 kDa), reside in the ONM. Smaller isoforms may be found in the INM [211, 212]. The large flexible cytoplasmic domains of Nesp1G and Nesp2G each feature an N-terminal ABD followed by multiple SRs. Nesp1G and Nesp2G localize to the ONM via their KASH domain, which binds in the NE lumen to the INM domain of SUN proteins. These nesprins are able to form a link to the actin cytoskeleton through their calponin homology domains [31, 213]. Nesprins are anchored in a similar way as SUN proteins but nesprin-3 interacts with IFs in the cytoplasm by binding to plectin [42]. Unlike nesprin-1 and -2, nesprin-3 lacks an ABD domain and is therefore unable to associate with actin directly, but forms an interconnected mesh with AFs, MTs, and IFs through plakin family members, plectin isoforms, and dystonin-a2 [43, 214–216], but it is unclear yet which isoform(s) is involved. Nesprin-3 has two splice variants, nesprin-3*α* and nesprin-3*β*. Only nesprin-3*α* contains an N-terminal binding site for plectin that provides a bridge to IF cytoskeletal elements via C-terminal of plectins (Figure 3) [42]. Nesprin-4 has been identified as a kinesin-1-binding protein in the NE [210] and nesprin-1 binds kinesin-2, mediating the transport of membranes to the midbody of cells undergoing cytokinesis [217]. These findings suggest that other nesprins at the NE may also bind kinesins. Interestingly, nesprin-4 expression seems to be restricted mainly to secretory epithelia, where it may influence centrosome/nuclear positioning and the relationship of the Golgi apparatus to the nucleus [210].

Proteins linking the NE and cytoskeleton have been the subject of many investigations (for reviews see [218, 219]). TorsinA has been shown to associate with a nesprin-3, plectin, and vimentin complex through its interaction with the KASH domain of nesprin [27]. The C-terminus of torsinA also associates with the KASH domain of nesprin-1 and nesprin-2. In the absence of torsinA, nesprin-3 moves away from the nucleus into the ER [27]. Also, in DYT1 patient fibroblasts nesprin-3 accumulates in globular structures, presumably derived from the NE/ER, suggesting that mutant torsinA interferes with the interaction of nesprin-3 with its binding partners in the NE [27]. We propose that both dystonin and torsinA influence the interaction between the NE and cytoskeleton through their association with nesprins in the ONM (Figure 3).

The association between dystonin-a2 and nesprin-3*α* highlights their role in linking the NE/ER to the cytoskeleton (Figure 3). The common modular structure between plectin isoforms and dystonin-a implicates the ABD as a critical mediator of interactions between multiple proteins and nesprin-3*α*. Two-hybrid analysis revealed that the ABD of dystonin-a facilitates interaction with nesprin-3*α* [42]. Furthermore, studies have elucidated the dystonin isoforms involved in this interaction, with dystonin-a2 (ABD and TM), but not isoform a3 (ABD only), being coimmunoprecipitated with nesprin-3*α* [41]. This association was attributed to the TM domain adjacent to the ABD at the N-terminus of dystonin-a2 that uniquely positions the protein near perikaryal membranes.

## 6. Conclusions

TorsinA and dystonin-a2 share many features, including causing neurologic diseases with a dystonic phenotype when mutated in humans and mice, respectively, with the onset of symptoms occurring at a young age. Although DYT1 dystonia is inherited as an autosomal dominant disease and *dt* dystonia in mice as an autosomal recessive disease, both represent a decrease or loss of function, respectively, of the normal wild-type protein. Both proteins are associated with the NE and ER. While torsinA remains in the contiguous lumen shared by the NE and ER, dystonin-a2 is a cytoplasmic protein associated with NE/ER, as well as Golgi membranes. Both torsinA and dystonin-a2 have been found to be associated with nesprin-3*α*, which forms a link between the ONM of the NE and the cytoskeleton, with mutated versions of both proteins causing abnormal morphology of the NE and formation of perinuclear inclusions involving cytoskeletal proteins. Loss of function of both proteins is associated with increased sensitivity to ER stress, which can cause cellular dysfunction and/or lead to neuronal apoptosis.

TorsinA and dystonin-a2 are both critical to neuronal functions, albeit in different neuronal subpopulations. Although DYT1 dystonia is primarily a disease of the brain without marked neurodegeneration and *dt* dystonia affects primarily peripheral sensory and motor neurons with neurodegeneration, there is evidence that some areas of the brain are also affected by *Dst* mutations. A distinct difference between diseases caused by mutations in these two proteins is the early death of *dt* mice, while DYT1 patients have few, if any, other medical problems related to their dystonia. Both proteins have been implicated in a variety of cellular processes, including processing of proteins through secretory pathways, transport of organelles along neuronal processes, and cell migration. Their broad involvement in cellular mechanisms can be explained by their interaction with the cytoskeleton, which is critical in many cellular processes. Thus, dystonin-a2 and torsinA share similar functions, and the reason they affect different neuronal populations presumably reflects their differential expression in specific neuronal subtypes. Dystonin-a2 is primarily expressed in sensory and motor neurons in the peripheral nervous system, whereas torsinA is mainly expressed in specific neurons in the CNS. Understanding their common functions supports the critical role of NE/ER cytoskeletal interactions in maintaining neuronal integrity and function, with dysfunction at many levels of the nervous system contributing to abnormal dystonic movements.

## Figures and Tables

**Table 1:** Mouse models of *dystonia. *

**Type/mutation**	**Anatomical phenotype**	**Motor phenotype**	**Reference**
Dystonin/Bpag1 mutations*			

*dt^24J^*, spontaneous	Nervous system	Hind limb clasping, dystonia	[175]
*dt^27J^*, spontaneous	Nervous system	Hind limb clasping, dystonia	[117]
*dt^alb^*, spontaneous	Behavior, nervous system, muscle	Dystonia	[176]
*dt^J^*, spontaneous	Mortality/aging, behavior, growth/size, pigmentation, vision/eye, hearing/vestibular/ear, integument	Abnormal posture, ataxia, impaired motor coordination	[177]
*dt^Tg4^*, transgenic (random gene disruption)	Mortality/aging, behavior, nervous system, muscle	Impaired motor coordination, hind limb clasping	[132]
*dt^tm1Efu^*, transgenic	Mortality/aging, nervous system, muscle, behavior, homeostasis, limbs/digits/tail, integument	Dystonia	[178]

DYT1 mutations^†^			

NSE-hMT, transgenic	Perinuclear aggregates stained for torsinA and ubiquitin	Hind limb clasping, marked hyperactivity, circling	[21]
CMV-hMT, transgenic	Not reported	Limited improvement on repeated rotarod testing in old animal	[165]
Prion-hMT, transgenic	Perinuclear aggregates stained for torsinA and ubiquitin	Limited improvement on repeated rotarod testing in old animals	[164]
torsinA knock-in, heterozygous	Perinuclear aggregates stained for torsinA and ubiquitin, NE abnormalities in neurons	Mild hyperactivity, poor performance on beam walking test	[23]
torsinA knock-out, heterozygous	Peduced amount of torsinA; NE abnormalities in neurons	Similar to knock-in(heterozygous)	[96]
Cortex-specific torsinA knock-out, heterozygous	Not reported	Deficiency on beam-walking test, hyperactivity	[98]
TH- hMT, transgenic	Not reported	Similar to CMV-hMT, transgenic	[104]

NSE: neuron-specific enolase promoter; CMV: cytomegalovirus promoter; hMT: human torsinA mutant; hWT: human torsinA wild type; NE: nuclear envelope; TH: tyrosine hydroxylase.

*Based on information retrieved from the International Mouse Genome Database (MGI)

^†^Adapted from Jinnah et al. [169].

## Abbreviations

AAA^+^: Superfamily of ATPases associated with diverse cellular activities
ATPases/proteins: Enzyme that catalyses the hydrolysis adenosine triphosphate
ABD: Actin-binding domain
AF: Actin filament
BP: Bullous pemphigoid
BPAG1: Bullous pemphigoid antigen 1
bpag1: Mouse bullous pemphigoid antigen 1
*C. elegans*: *Caenorhabditis elegans*
CNS: Central nervous system
DA: Dopamine
Δ*E*: Deletion of glutamic acid
*DST*: Human dystonin gene
*Dst*: Mouse dystonin gene
*dt*: *Dystonia musculorum*
DYT1: Early-onset, torsion dystonia
EB1: Microtubule end-binding protein 1
ER: Endoplasmic reticulum
ERAD: ER-associated degradation
Gas2: growth arrest-specific 2
IF: Intermediate filament
IFBD: IF-binding domain
INM: Inner nuclear membrane
Hypb: Hydrophobic amino terminal domain
KASH: Klarsicht, ANC-1, Syne homology
LAP1: Lamina-associated polypeptide 1
LINC: Linker of nucleoskeleton and cytoskeleton complex
LULL1: Lumenal domain like LAP1
MACF1: Microtubule actin cross-linking factor 1
MEFs: Mouse embryonic fibroblasts
MT: Microtubule
MTBD: MT binding domain
myr: Myristylation motif
NE: nuclear envelope
Nesp1G: Nesprin-1 Giant
Nesp2G: Nesprin-2 Giant
nesprins: Nuclear envelope spectrin repeat
NF: Neurofilament
ONM: Outer nuclear membrane
PDI: Protein disulfide isomerase
SGCE: *ε*-sarcoglycan
SS: Signal sequence
SRs: Spectrin repeats
SUN1: Sad1 and UNC84 domain containing 1
TM: Transmembrane domain
*TOR1A; *previously referred to as *DYT1*: human gene encoding torsinA
*TOR2*: Torsin2 gene
*TOR3*: Torsin3 gene
VIMP: VCP-interacting membrane protein.
